# Occult Incarcerated Osteochondral Fracture of the Medial Patellar Facet Following Acute Lateral Patellar Dislocation in an Adolescent: A Case Report

**DOI:** 10.7759/cureus.116564

**Published:** 2026-09-19

**Authors:** Laith Hseinat, Omar Al Qudah, Yazan Albatayneh, Saad Alkayid, Hamza A Abuain, Yasmeen Foda, Mutaz Jaloody, Ashraf Albadaineh

**Affiliations:** 1 Orthopaedics, Jordanian Royal Medical Services, Amman, JOR; 2 General Practice, Princess Basma Hospital, Irbid, JOR; 3 Orthopaedics and Traumatology, Ministry of Health, Amman, JOR

**Keywords:** adolescent, headless compression screw, incarcerated osteochondral fragment, medial patellar facet, medial patellofemoral ligament (mpfl), osteochondral fracture, patellar dislocation

## Abstract

Acute lateral patellar dislocation is a common injury in adolescents and is most frequently associated with rupture of the medial patellofemoral ligament (MPFL). Concomitant osteochondral fractures may remain occult or be misinterpreted on initial imaging, resulting in delayed diagnosis and potentially compromising long-term joint function. We present a rare case of an incarcerated osteochondral fracture of the medial patellar facet that was initially misinterpreted as arising from the medial femoral condyle following acute lateral patellar dislocation.

A previously healthy 15-year-old boy presented to King Hussein Medical City Emergency Department approximately one week after his injury following an acute traumatic lateral patellar dislocation sustained while playing football. Clinical examination demonstrated persistent lateral patellar subluxation and knee instability. Initial plain radiographs and computed tomography demonstrated a displaced osteochondral fragment and persistent lateral patellar subluxation; however, the fragment was initially interpreted as arising from the medial femoral condyle. Although advanced imaging was performed, the anatomical origin of the incarcerated osteochondral fragment was not correctly recognized preoperatively. Surgical exploration unexpectedly revealed an incarcerated osteochondral fracture of the medial patellar facet rather than an isolated ligamentous injury. The fragment was anatomically reduced and stabilized using three headless compression screws supplemented with a suture anchor to repair the MPFL, successfully restoring the patellar articular surface and patellar stability. A structured postoperative rehabilitation program resulted in fracture healing, progressive functional recovery, and return to pre-injury activities without recurrent patellar instability.

This case highlights the diagnostic challenge of occult osteochondral injuries associated with acute lateral patellar dislocation in adolescents. Persistent patellar maltracking or subluxation following reduction should raise suspicion for an incarcerated osteochondral fragment, even when conventional imaging appears consistent with an isolated MPFL injury. Careful preoperative evaluation combined with meticulous intraoperative assessment is essential for identifying repairable osteochondral lesions. Early anatomical reduction and stable fixation can restore joint congruity, preserve native articular cartilage, and achieve excellent functional outcomes while potentially reducing the risk of recurrent instability and post-traumatic osteoarthritis.

## Introduction

Acute patellar dislocation is a common knee injury, particularly among children, adolescents, and young active individuals. It is an important cause of traumatic knee hemarthrosis and accounts for approximately 2%-3% of knee injuries [1]. The incidence is highest during the second decade of life, with many first-time dislocations occurring during sporting activities or other physical activities involving sudden changes in direction, valgus loading, or rotational forces. The vast majority of acute patellar dislocations occur laterally, reflecting disruption of the complex static and dynamic stabilizing mechanisms of the patellofemoral joint [1].

The patellofemoral joint is formed by the articulation of the patella with the femoral trochlea. The medial patellar facet forms part of the medial articular surface of the patella, whereas the femoral trochlea provides the bony track along which the patella engages during knee flexion. The medial patellofemoral ligament (MPFL) is an important medial soft-tissue stabilizer that helps prevent lateral displacement of the patella [2].

Among the medial soft-tissue stabilizers, the MPFL plays a central role in preventing lateral displacement of the patella, providing a substantial proportion of the restraining force against lateral patellar translation, particularly during early knee flexion [3]. Consequently, MPFL injury is frequently associated with acute lateral patellar dislocation and represents a characteristic component of the injury pattern. However, acute patellar dislocation should not be considered an isolated ligamentous injury, as substantial intra-articular damage may occur simultaneously, including injury to the articular cartilage, subchondral bone, and osteochondral surface [4].

Chondral and osteochondral injuries are important associated injuries following acute lateral patellar dislocation, particularly in children and adolescents. During the dislocation and subsequent reduction of the patella, shearing forces may produce damage to the medial patellar facet, the lateral femoral condyle, or both. These injuries may range from superficial chondral defects to displaced osteochondral fractures and intra-articular loose bodies. In addition to causing persistent pain, mechanical symptoms, and functional limitation, untreated osteochondral lesions may compromise articular congruity and potentially contribute to progressive cartilage degeneration and early patellofemoral osteoarthritis [5,6].

Chondral and osteochondral injuries following acute patellar dislocation have been classified into three groups: cracks alone, cartilage defects caused by osteochondral or chondral fractures, and cartilage defects caused by osteochondral or chondral fractures associated with cracks [7].

Recent evidence has further emphasized the frequency of these associated injuries. A systematic review and meta-analysis involving more than 3,300 patients reported an overall prevalence of osteochondral injury of approximately 49% following acute lateral patellar dislocation, with the medial patella representing the most frequently involved site [8]. Similarly, MRI-based studies in pediatric populations have demonstrated that osteochondral fractures and other clinically relevant intra-articular injuries are common after first-time patellar dislocation, with the medial patellar facet being a particularly frequent location [9,10]. These findings highlight the importance of careful evaluation of the articular surfaces following an acute patellar instability event.

Despite their clinical importance, osteochondral fractures may be overlooked during the initial assessment. Plain radiographs may fail to identify predominantly cartilaginous fragments or small osteochondral lesions, particularly when the osseous component is minimal [10]. According to previous research, the detection rate of osteochondral fragments associated with acute patellar dislocation on conventional radiographs is significantly lower than that reported by surgical or arthroscopic findings. CT, particularly when combined with multiplanar reconstruction, may provide a more accurate assessment of these injuries by offering improved visualization and characterization of the size and location of bony fragments [11]. Even advanced imaging may occasionally fail to clearly demonstrate the true morphology or position of an osteochondral fragment, especially when it is displaced, overlapped with adjacent structures, or incarcerated within the patellofemoral articulation. Previous studies have demonstrated that a substantial proportion of osteochondral injuries following acute patellar dislocation may not be recognized on initial radiographic assessment [10]. A systematic review evaluating imaging modalities for osteochondral fractures following traumatic patellar dislocation further emphasized the important role of advanced imaging, particularly MRI, in identifying associated cartilage injuries and loose bodies and assisting surgical decision-making [12].

Early recognition of a repairable osteochondral fracture is clinically important because preservation and anatomical fixation of a sufficiently large viable fragment may restore the native articular surface and avoid the consequences associated with fragment excision or untreated cartilage loss. Conversely, a missed incarcerated fragment may contribute to persistent patellar maltracking, recurrent subluxation, mechanical symptoms, and ongoing articular damage. Therefore, persistent patellar malalignment or subluxation after an apparent reduction should prompt careful reassessment for an associated intra-articular or osteochondral lesion, even when the initial imaging findings appear to suggest a predominantly ligamentous injury.

We report the case of a 15-year-old boy who sustained an acute traumatic lateral patellar dislocation associated with an occult incarcerated osteochondral fracture of the medial patellar facet and concomitant MPFL injury. Initial radiographic and computed tomography (CT) findings suggested persistent patellar subluxation associated predominantly with MPFL injury, while the osteochondral fragment remained unrecognized preoperatively. Surgical exploration revealed an incarcerated medial patellar facet osteochondral fracture, which was anatomically reduced and fixed, followed by repair of the associated MPFL injury. This case highlights an unusual diagnostic pitfall following acute patellar dislocation and emphasizes the importance of maintaining a high index of suspicion for occult osteochondral injury when persistent patellar malalignment cannot be fully explained by an isolated soft-tissue injury.

## Case presentation

A 15-year-old previously healthy boy presented to our tertiary orthopedic center with persistent right knee pain, swelling, restricted knee motion, and inability to bear weight following a traumatic injury sustained while playing football. The injury occurred one week before presentation when the patient fell and sustained a direct blow to the anterior aspect of his right knee. He experienced immediate pain and swelling associated with a sensation of patellar instability. These findings were not specific for an incarcerated osteochondral fragment and could also be observed in an isolated MPFL injury.

The patient was initially evaluated at a peripheral hospital, where plain radiographs reportedly showed no obvious fracture. An above-knee posterior slab was applied for immobilization and symptomatic management. Four days later, he was reviewed at an orthopedic clinic, where continued conservative management was recommended without further advanced imaging. Because of persistent symptoms and concerns regarding the diagnosis, the family sought a further opinion, and the patient subsequently presented to our tertiary orthopedic center one week after the initial injury.

On presentation, physical examination demonstrated moderate swelling of the right knee with localized tenderness over the medial aspect of the patella. The injury was closed, and distal neurovascular examination was normal. Passive knee range of motion was preserved but painful, whereas active motion was limited by pain and apprehension. The patient was unable to bear weight, and a positive patellar apprehension test raised concern for persistent lateral patellar instability.

Plain radiographs demonstrated a rim-like osseous fragment projected adjacent to the medial femoral condyle, raising suspicion of an associated osseous avulsion injury (Figure 1). CT confirmed the presence of the fragment and demonstrated persistent lateral patellar subluxation, without other obvious fractures (Figure 2). MRI was not performed during the preoperative evaluation. The initial CT images were reviewed by the treating team, including a senior specialist, a junior specialist, and a resident. The images demonstrated a displaced osseous fragment overlying the medial femoral condyle, raising suspicion for an associated patellar or medial femoral condyle injury. The CT study was of suboptimal quality because of motion artifacts, which limited confident assessment of the fragment’s anatomical origin. Following surgery, the preoperative CT images were retrospectively reviewed by a senior orthopedic specialist and a musculoskeletal radiologist, with knowledge of the intraoperative findings. On retrospective review, the fragment was identified as originating from the medial patellar facet (Figures 3-4). A repeat CT was avoided because of the patient’s young age and concern about additional radiation exposure. Given the persistent patellar subluxation, surgical exploration was performed with preparation for osteochondral fragment fixation if identified. Based on the clinical and radiological findings, the patient was diagnosed with acute traumatic lateral patellar instability associated with a suspected medial-sided injury and was admitted for definitive surgical management.

**Figure 1:**
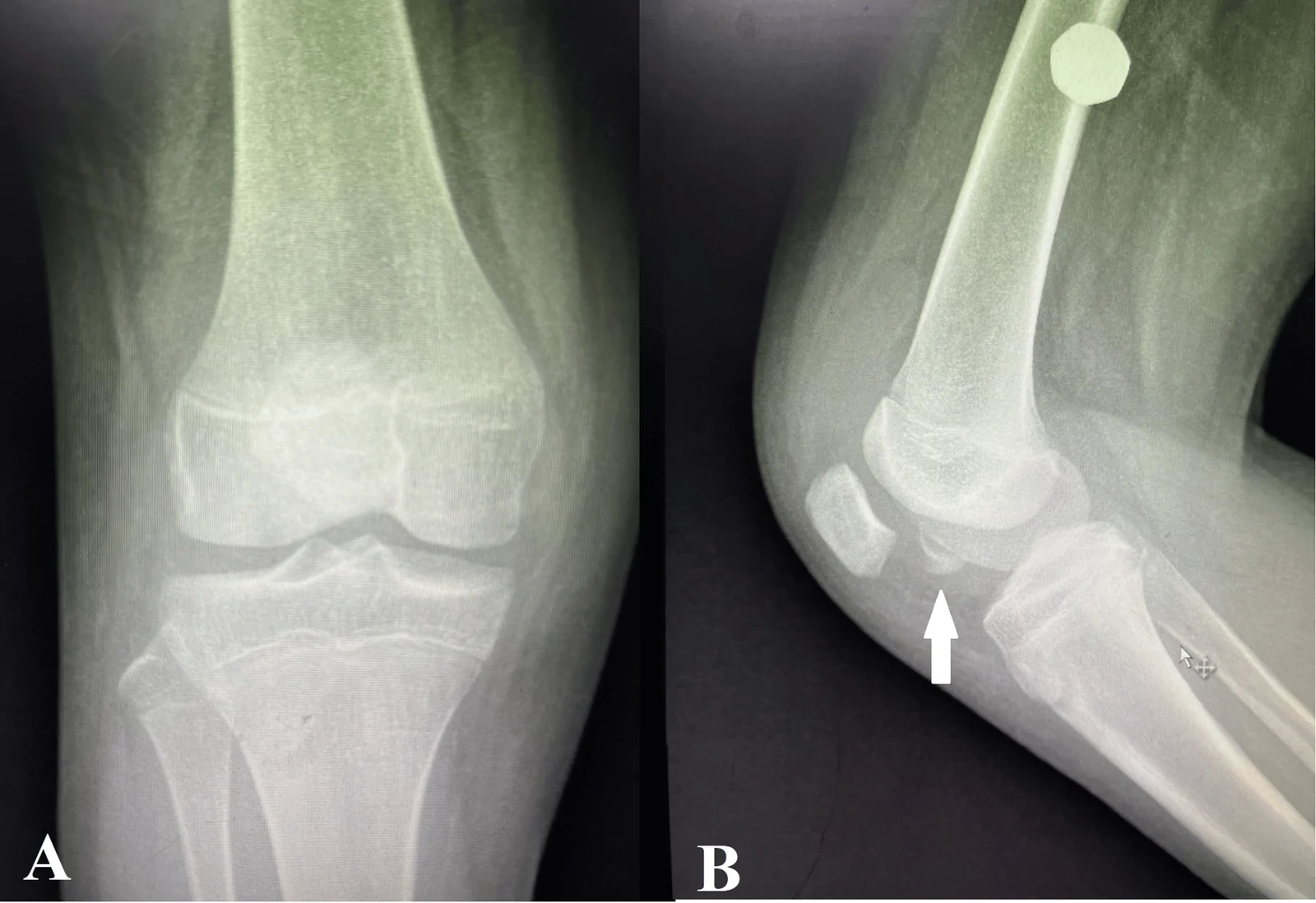
Initial Plain Radiographs of the Right Knee Demonstrating an Osteochondral Fragment (A,B) Initial plain radiographs of the right knee, including anteroposterior (A) and lateral (B) views, demonstrating preserved knee alignment without an obvious patellar fracture or other acute osseous abnormality. A small displaced osteochondral fragment is visible projecting over the medial femoral condyle (arrow).

**Figure 2:**
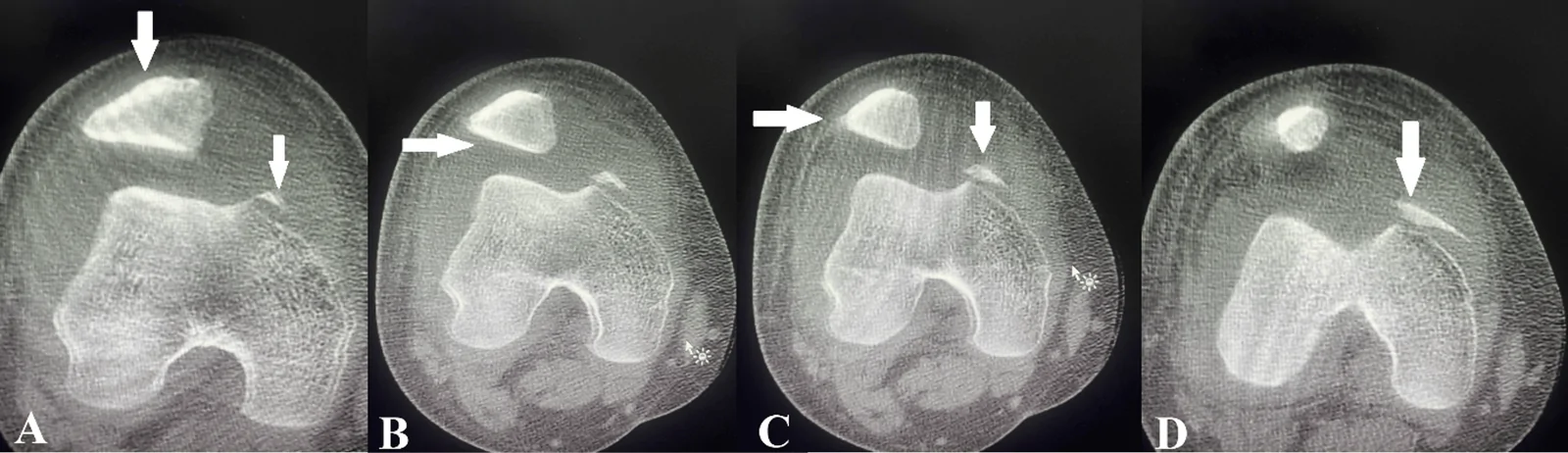
Axial CT Images of the Right Knee Demonstrating Patellar Subluxation and an Osteochondral Fragment (A-D) Axial computed tomography (CT) images of the right knee demonstrating persistent lateral patellar subluxation (arrow) and significant hemarthrosis. A displaced osteochondral fragment is identified within the joint, projecting over the medial femoral condyle (arrow), which was initially interpreted as originating from the medial femoral condyle.

**Figure 3:**
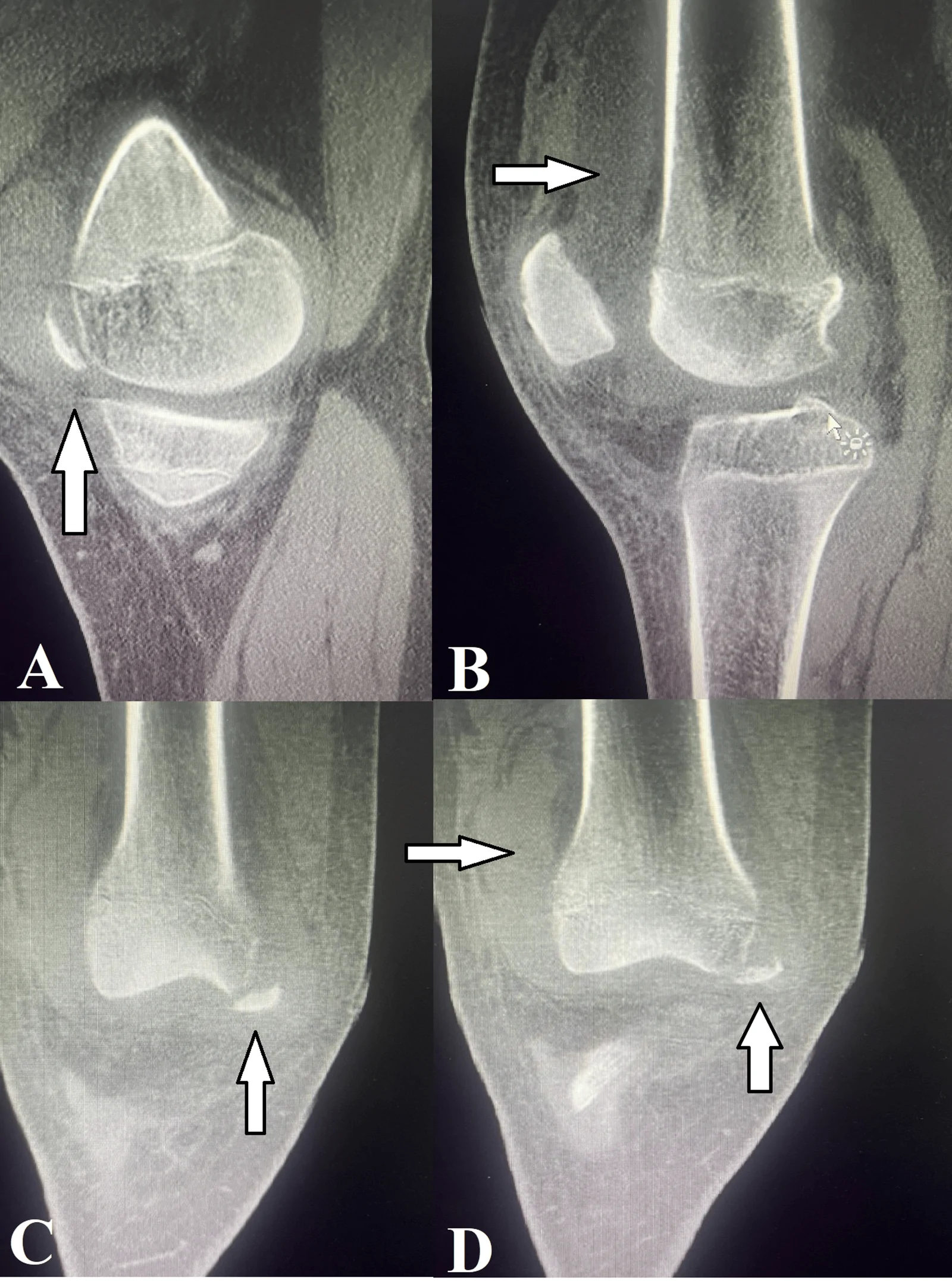
Sagittal and Coronal CT Images of the Right Knee Demonstrating Hemarthrosis and a Displaced Osteochondral Fragment (A,B) Sagittal computed tomography (CT) images of the right knee demonstrating significant intra-articular hemarthrosis (arrow) and a displaced osteochondral fragment (arrowhead). (C,D) Coronal CT images further demonstrating the intra-articular osteochondral fragment (arrow) associated with significant hemarthrosis (arrow).

**Figure 4:**
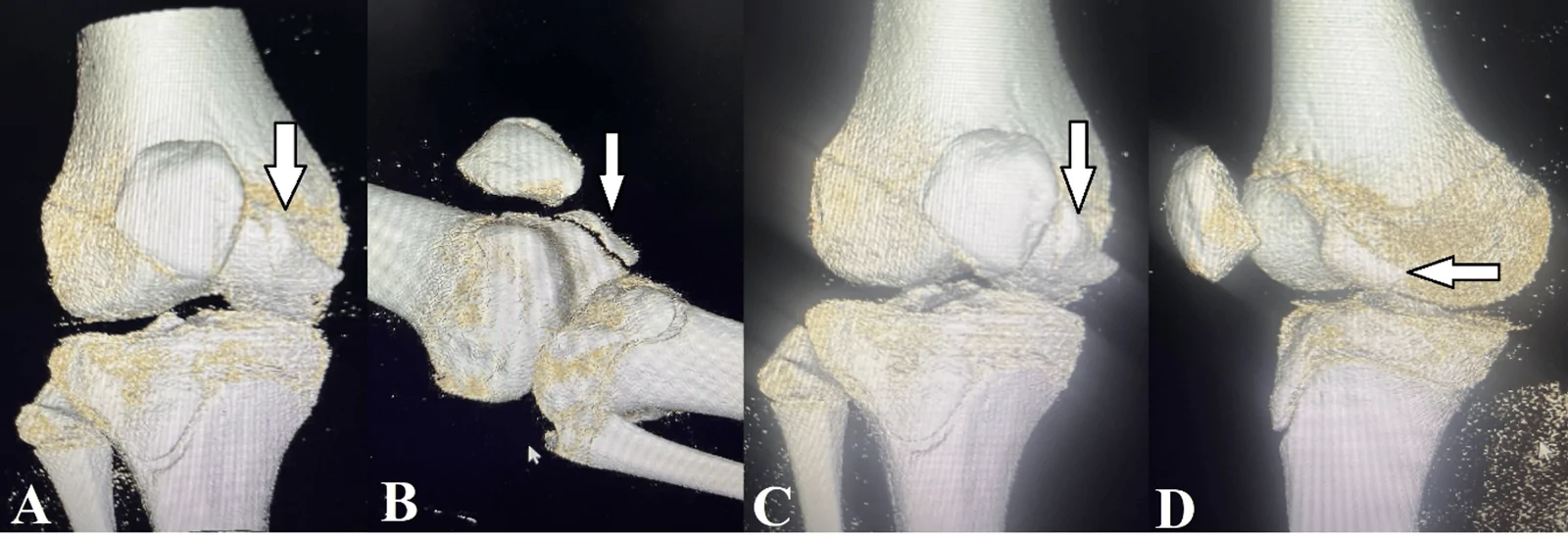
Three-Dimensional CT Reconstruction of the Right Knee Demonstrating a Displaced Osteochondral Fragment (A-D) Three-dimensional computed tomography (3D CT) reconstruction of the right knee demonstrating a displaced osteochondral fragment projecting over the medial femoral condyle (arrow). The fragment was initially interpreted as originating from the medial femoral condyle, with no obvious patellar osseous abnormality identified on preoperative imaging.

Surgical intervention was performed on the second day of admission. Under spinal anesthesia, with the patient in the supine position, a 6 cm midline anterior knee incision followed by a medial parapatellar arthrotomy was performed. A significant intra-articular hematoma was encountered and evacuated. Intraoperative exploration revealed a displaced and incarcerated osteochondral fragment originating from the medial patellar facet, which had not been correctly identified during the preoperative imaging assessment (Figure 5).

**Figure 5:**
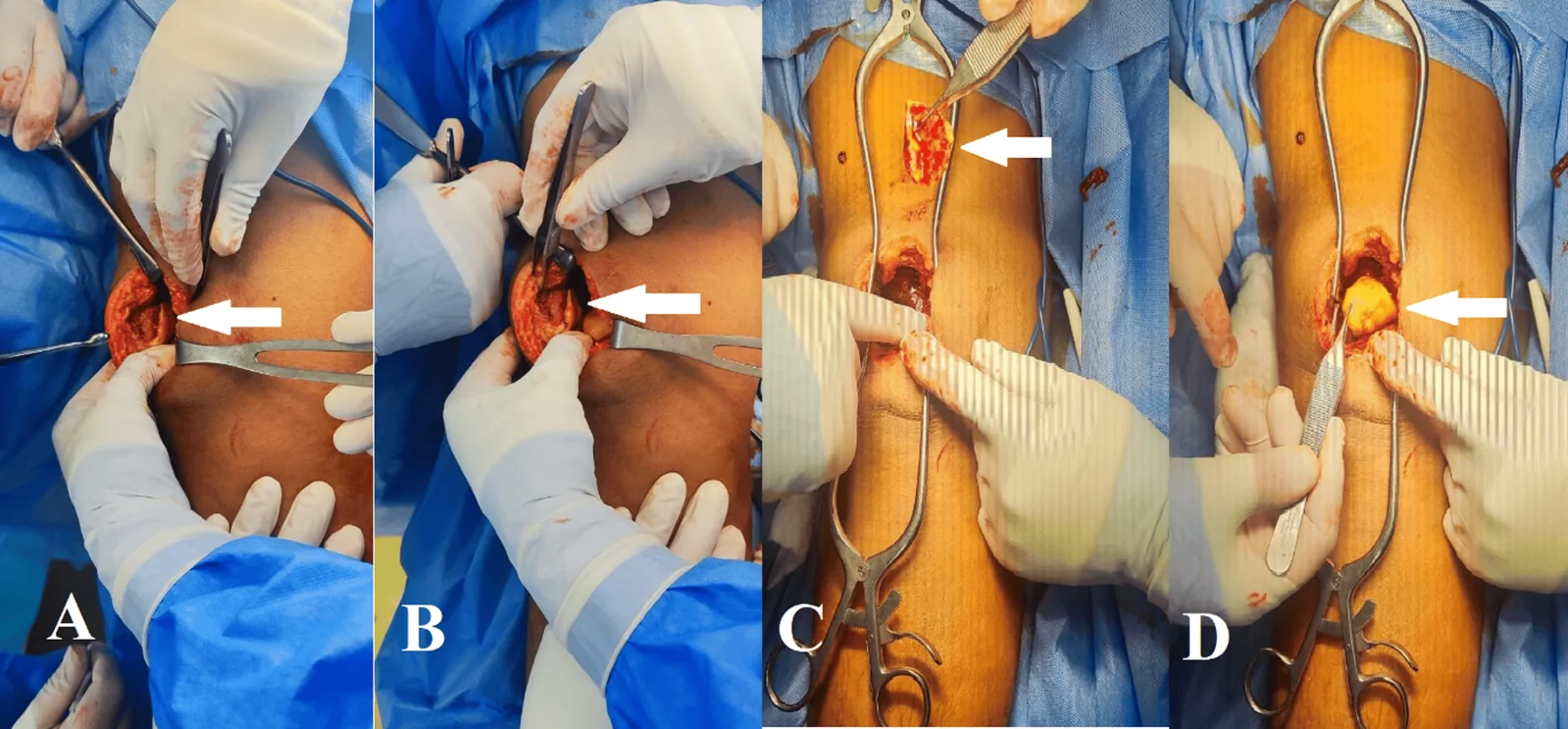
Intraoperative Findings and Retrieval of the Displaced Osteochondral Fragment (A,B) Intraoperative photographs demonstrating a large osteochondral defect involving the medial patellar facet (arrows), confirming the site of origin of the displaced osteochondral fragment. (C,D) Intraoperative photographs showing the retrieved large osteochondral fragment, measuring approximately 2 × 3 cm (arrows).

The osteochondral fragment was carefully retrieved and anatomically reduced to its native position on the medial patellar facet. The fragment measured approximately 2-2.5 × 3-3.5 cm, with a subchondral bone thickness of approximately 2-3 mm that varied across different areas of the fragment. It was provisionally stabilized using three 1.0-mm Single Trocar Kirschner wires (Rita Leibinger Medical, Mühlheim an der Donau, Germany), followed by definitive fixation with three 2.5 × 26-mm Herbert headless compression screws (Jiangsu Baide Medical Instrument Co., Ltd., Zhangjiagang, China), restoring the congruity of the patellar articular surface (Figure 6). Following fixation of the osteochondral fragment, the patella remained unstable, with persistent lateral instability on intraoperative assessment. Therefore, repair of the medial patellofemoral ligament (MPFL) was performed using a 5.0-mm HealFix titanium suture anchor (Hangzhou Rejoin Medical Group Co., Ltd., Hangzhou, China) to restore medial patellar stability. Patellar tracking and stability were reassessed following repair and found to be satisfactory (Figure 7). The wound was thoroughly irrigated and closed in layers. The knee was initially immobilized in an above-knee posterior slab, which was replaced with a hinged knee brace on the first postoperative day.

**Figure 6:**
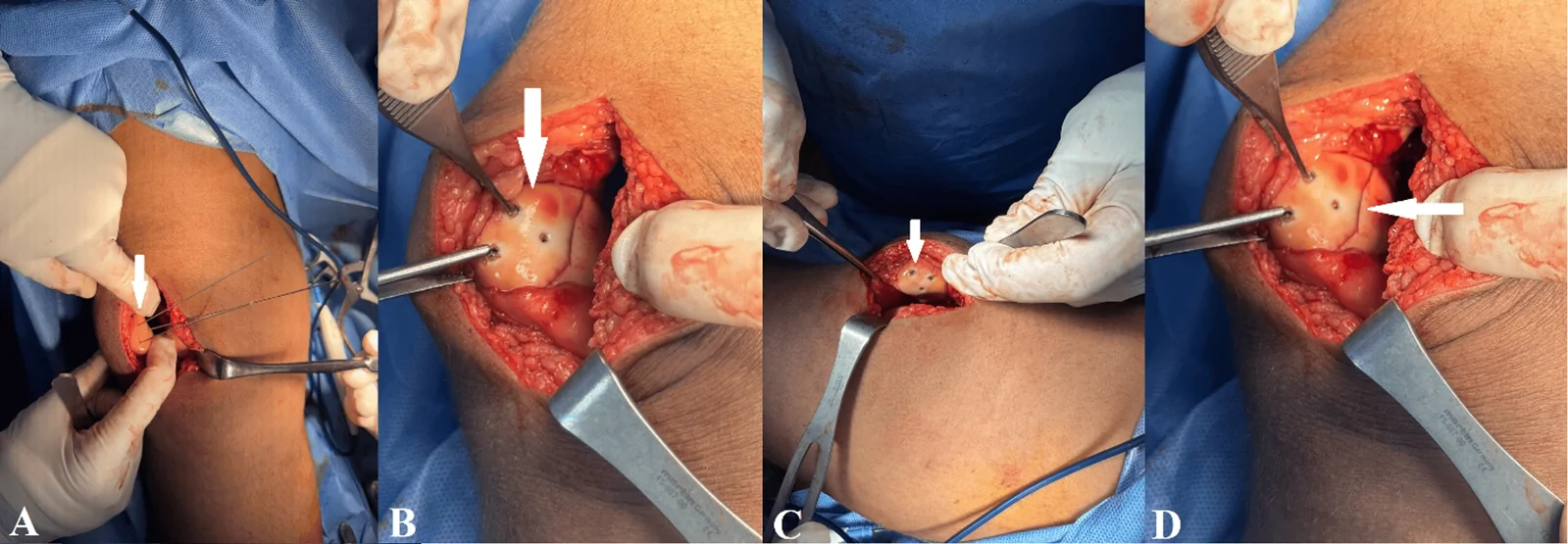
Intraoperative Reduction and Fixation of the Osteochondral Fragment of the Medial Patellar Facet (A) Intraoperative photograph demonstrating anatomical reduction of the osteochondral fragment into the defect of the medial patellar facet, followed by provisional fixation with Kirschner wires (arrow). (B-D) Intraoperative photographs demonstrating the restored articular congruity following anatomical reduction of the osteochondral fragment and definitive fixation with headless compression screws (arrows).

**Figure 7:**
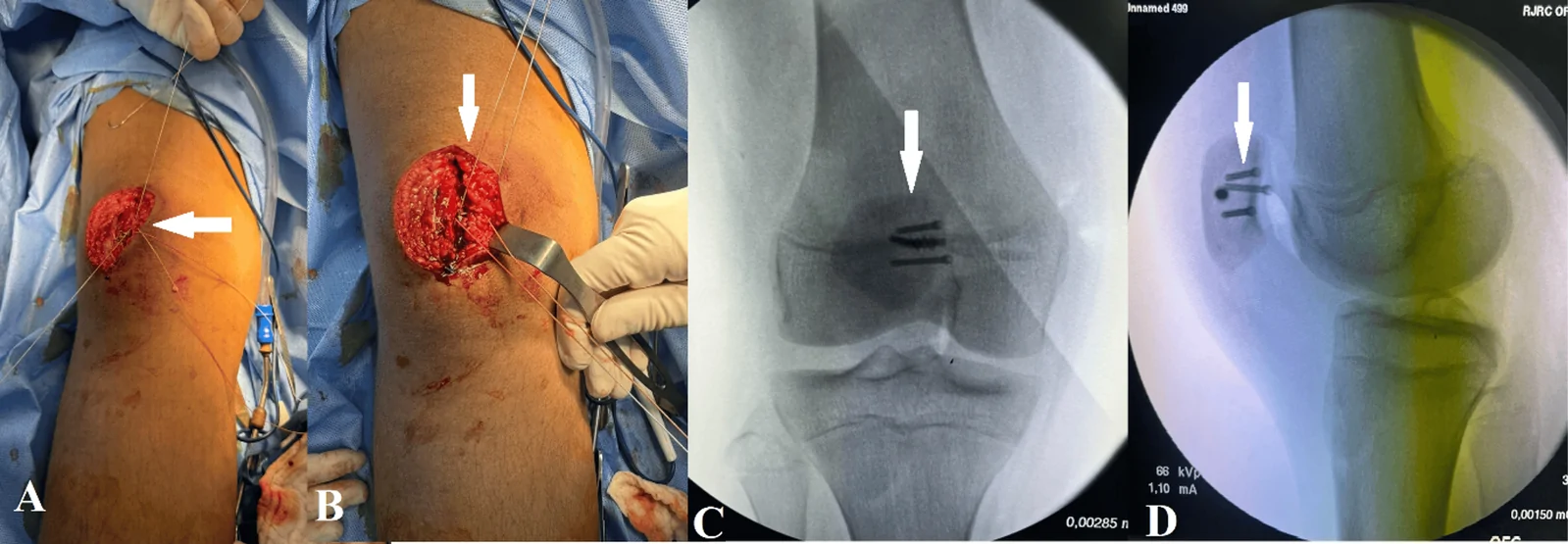
Intraoperative Repair of the Medial Patellofemoral Ligament and Fixation of the Osteochondral Fragment (A,B) Gross intraoperative photographs demonstrating repair of the medial patellofemoral ligament (MPFL) using a suture anchor placed at the medial aspect of the patella and secured to the medial retinacular tissues to restore patellar stability (arrows). (C,D) Intraoperative fluoroscopic anteroposterior (C) and lateral (D) views demonstrating anatomical reduction of the osteochondral fragment within the medial patellar facet defect, restoration of patellar articular surface congruity, and the final position of the three headless compression screws and patellar suture anchor (arrows).

Postoperatively, the patient followed a structured rehabilitation protocol. The knee was maintained in a hinged brace at 30° of flexion for four weeks, with adequate analgesia and pharmacological thromboprophylaxis using Innohep 4500 IU subcutaneously once daily for four weeks. Progressive range-of-motion exercises were subsequently initiated, with the permitted flexion increased by approximately 15° each week. Progressive weight-bearing was allowed as tolerated. A structured strengthening program was introduced following progression of range of motion and clinical recovery (Figure 8).

**Figure 8:**
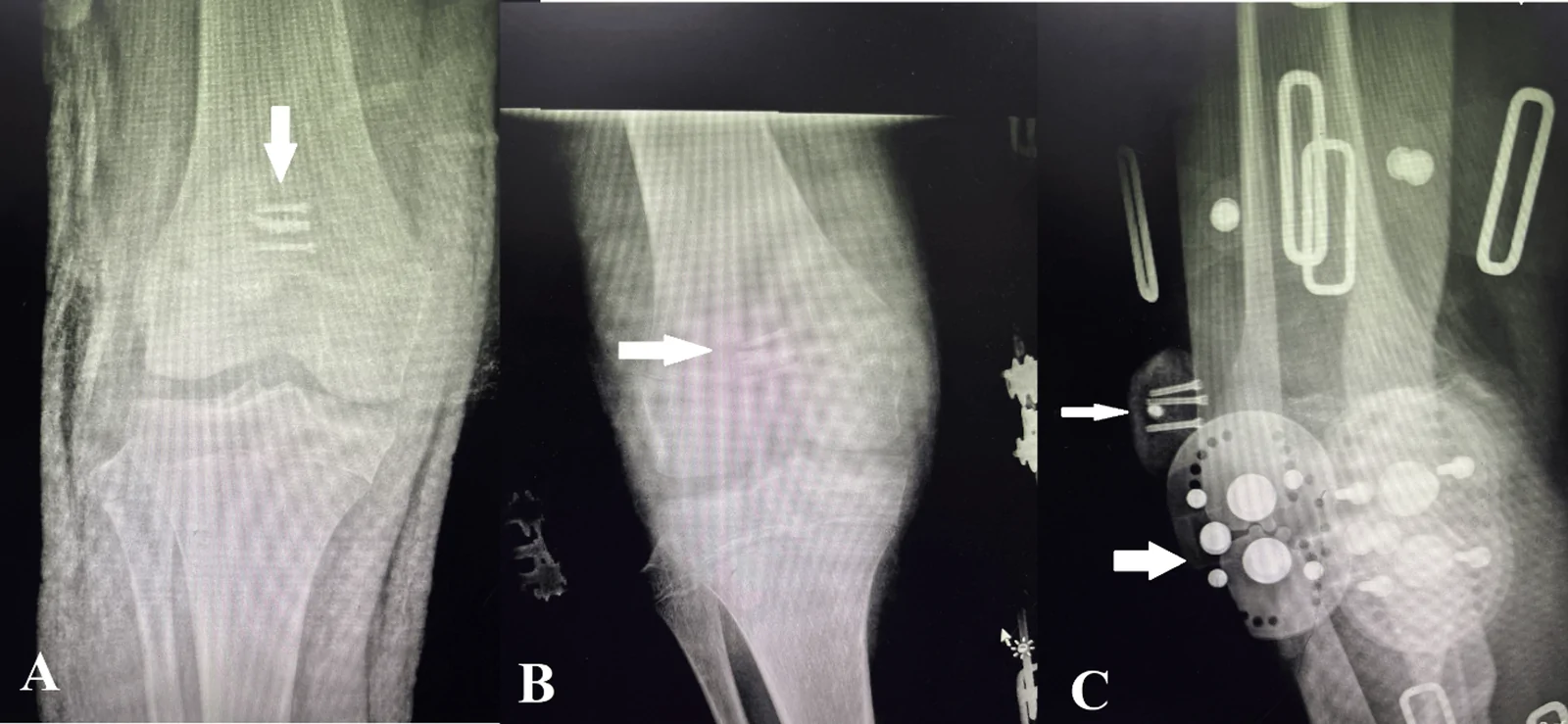
Postoperative Radiographic Evaluation of Osteochondral Fragment Fixation and Patellar Alignment (A) Immediate postoperative anteroposterior radiograph of the right knee demonstrating anatomical reduction and fixation of the osteochondral fragment, with appropriate positioning of the three headless compression screws and the patellar suture anchor. The patella is centrally aligned within the trochlear groove. (B,C) Eight-week follow-up anteroposterior (B) and lateral (C) radiographs demonstrating maintained central patellar alignment and stable positioning of the headless compression screws and suture anchor, without peri-implant radiolucency or evidence of fixation failure (arrows). A hinged knee brace was used during the early rehabilitation period to protect the repair while allowing progressive knee mobilization.

At the five-month follow-up, the patient demonstrated satisfactory wound healing and progressive functional recovery. At five-month follow-up, he had achieved approximately 110° of knee flexion and was able to walk independently and return to school without significant limitation. This represented a residual limitation in knee flexion, although no recurrent patellar instability was noted. The patellar apprehension test was negative, with no clinical evidence of recurrent patellar instability. Radiographic evaluation demonstrated maintained reduction of the osteochondral fragment with stable implant positioning.

## Discussion

Acute lateral patellar dislocation commonly affects adolescents and young, physically active individuals, particularly during sporting activities. In addition to disruption of the medial stabilizing structures, first-time patellar dislocation may be associated with chondral or osteochondral injuries that can significantly influence both short- and long-term clinical outcomes. Failure to recognize these associated injuries may result in persistent symptoms, articular incongruity, recurrent instability, and potentially premature degenerative changes. Therefore, early identification and appropriate management of associated osteochondral lesions are essential to restore joint congruity and preserve the native articular surface [13].

The mechanism of injury typically involves lateral displacement of the patella across the lateral femoral trochlea, often in association with valgus and rotational forces acting on a partially flexed knee. During dislocation and subsequent reduction, substantial compressive and shear forces may develop between the medial patellar facet and the lateral femoral condyle. Consequently, osteochondral injury most commonly involves the medial patellar facet and/or the lateral femoral condyle [14]. This mechanism is consistent with the injury pattern observed in our patient, who sustained a traumatic sports-related injury and was subsequently found to have an osteochondral fracture originating from the medial patellar facet.

The MPFL represents the principal passive restraint against lateral patellar translation during early knee flexion, providing approximately 50%-60% of the resistance to lateral displacement before the osseous constraints of the trochlea become increasingly important. Injury to the MPFL is common following a first-time lateral patellar dislocation and may contribute to persistent or recurrent instability when medial soft-tissue restraint is not adequately restored [15]. In the present case, although the exact morphology and location of the MPFL injury were not definitively characterized intraoperatively, persistent patellar instability remained after anatomical fixation of the osteochondral fragment. Therefore, MPFL repair was performed to restore medial restraint and achieve satisfactory patellar stability.

Diagnosis of osteochondral injury following acute patellar dislocation remains challenging. Plain radiographs may fail to identify osteochondral fragments, particularly when fragments are small, predominantly cartilaginous, or superimposed on adjacent osseous structures. Advanced imaging can improve detection and characterization of associated intra-articular injuries; however, diagnostic errors may still occur when the anatomical origin or orientation of an osteochondral fragment is not carefully assessed [9]. In our patient, the osteochondral fragment was visible on preoperative CT imaging but was initially interpreted as arising from the medial femoral condyle. Retrospective assessment demonstrated that the fragment originated from the medial patellar facet. This discrepancy highlights an important diagnostic pitfall: the presence of an apparently isolated medial-sided osseous fragment in association with persistent patellar subluxation should prompt careful assessment of both the patellar and femoral articular surfaces.

An additional notable feature of this case was the incarceration of the osteochondral fragment within the patellofemoral joint. The persistent lateral patellar subluxation and failure to restore normal patellofemoral alignment raised concern for an underlying intra-articular mechanical component in addition to the associated MPFL injury. Surgical exploration ultimately revealed the incarcerated medial patellar facet fragment, which had not been correctly identified preoperatively. This finding emphasizes that persistent maltracking or subluxation following an acute patellar dislocation should not automatically be attributed solely to ligamentous injury. An incarcerated osteochondral fragment should be considered, particularly when imaging findings do not fully explain the degree of persistent deformity or instability.

Patellar osteochondral fractures are particularly important in children and adolescents because delayed diagnosis may compromise preservation of viable articular cartilage and adversely affect long-term joint function. When a sufficiently large and viable osteochondral fragment is present, preservation and anatomical fixation are generally preferred over fragment excision whenever stable reconstruction is technically feasible. Restoration of the articular surface may improve joint congruity and preserve native cartilage, whereas fragment excision may result in a persistent articular defect and increase the potential for subsequent degenerative changes [16].

Several fixation techniques have been described for repairable osteochondral fractures, including suture-based fixation, bioabsorbable implants, and headless compression screws. The choice of fixation should be individualized according to fragment size, location, cartilage integrity, and the amount of attached subchondral bone [6]. Headless compression screws can provide stable interfragmentary compression while minimizing implant prominence at the articular surface, making them a useful option for large osteochondral fragments with sufficient osseous thickness.

In the present case, the displaced osteochondral fragment was sufficiently large and viable to permit anatomical reconstruction. The fragment was reduced to its native position on the medial patellar facet, provisionally stabilized, and definitively fixed using three Herbert headless compression screws. This restored the congruity of the patellar articular surface. However, fixation of the osteochondral fragment alone did not restore satisfactory patellar stability. Persistent lateral instability following reduction prompted intraoperative MPFL repair using a suture anchor. This sequential approach allowed restoration of both the articular surface and medial patellar stability without relying solely on the preoperative imaging diagnosis.

At five months of follow-up, the patient had achieved approximately 110° of knee flexion, was able to walk independently and return to school, and demonstrated a negative patellar apprehension test without evidence of recurrent instability. Radiographic evaluation demonstrated maintained reduction of the osteochondral fragment and stable implant positioning. Although longer follow-up is necessary to evaluate cartilage healing, implant-related complications, return to sport, and the potential development of patellofemoral degenerative changes, the early outcome supports anatomical preservation and stable fixation of a repairable osteochondral fragment combined with restoration of patellar stability.

This case has limitations related to retrospective imaging review. The CT was reassessed after the intraoperative diagnosis was known, so hindsight bias cannot be excluded. Additionally, motion artifacts limited CT quality and assessment of the fragment’s origin, and MRI was not performed, which may have provided additional characterization of the osteochondral and soft-tissue injuries. Overall, this case highlights an important diagnostic challenge following acute lateral patellar dislocation in adolescents. Osteochondral fragments may be visible on advanced imaging yet remain misinterpreted regarding their anatomical origin. Persistent patellar maltracking or subluxation associated with an unexplained medial-sided osseous fragment should raise suspicion for an incarcerated osteochondral fracture. Careful reassessment of preoperative imaging and meticulous intraoperative evaluation are therefore essential. When a viable osteochondral fragment is identified, anatomical reduction and stable fixation, combined with restoration of patellar stability when required, may preserve the native articular surface and provide favorable early functional recovery.

## Conclusions

Acute lateral patellar dislocation in adolescents may be associated with clinically significant osteochondral fractures that can be overlooked or misinterpreted on initial imaging. This case highlights a diagnostic pitfall in which an incarcerated osteochondral fracture of the medial patellar facet was visible on initial radiographs and CT but was initially attributed to the medial femoral condyle. Persistent patellar maltracking or instability following acute dislocation should prompt careful reassessment for associated intra-articular pathology. Thorough intraoperative evaluation remains essential when imaging findings do not fully explain the clinical presentation. At five-month follow-up, anatomical fixation of the viable osteochondral fragment combined with MPFL repair resulted in favorable early functional recovery and maintained radiographic reduction. Longer-term follow-up is required to determine the durability of joint congruity and the potential impact on post-traumatic osteoarthritis.
